# IL-22-Expressing Murine Lymphocytes Display Plasticity and Pathogenicity in Reporter Mice

**DOI:** 10.3389/fimmu.2015.00662

**Published:** 2016-01-19

**Authors:** Wei Shen, Julie A. Hixon, Mairi H. McLean, Wen Qing Li, Scott K. Durum

**Affiliations:** ^1^Cancer and Inflammation Program, Laboratory of Molecular Immunoregulation, Center for Cancer Research, National Cancer Institute, National Institutes of Health, Frederick, MD, USA

**Keywords:** IL-22, Th17, Th22, T cell plasticity, IL-27

## Abstract

IL-22 has multiple activities ranging from tissue repair to inflammation. To characterize the pathogenicity and plasticity of cells that produce IL-22, a novel reporter mouse strain was generated. Homeostatic IL-22 reporter expression was observed in intestinal lymphoid cells identified as CD4 T cells and ILC3 cells. In a model of inflammatory bowel disease, CD4 T cells strongly expressed the IL-22 reporter in mesenteric lymph node. To examine plasticity of IL-22^+^ T cells, they were purified after generation *in vitro* or *in vivo* from inflamed colon, and then cultured under Th1, Th2, or Th17 conditions. *In vitro*-generated IL-22^+^ CD4 T cells showed relatively durable IL-22 expression under Th1 or Th2 conditions, whereas *in vivo*-generated cells rapidly lost IL-22 expression under these conditions. *In vitro*-generated cells could not be diverted to express Th1 or Th2 cytokines despite the expression of “master regulators.” *In vivo-*generated cells could be diverted, at very low frequency, to express Th1 or Th2 cytokines. Both *in vitro*- and *in vivo*-generated cells could be induced *in vitro* to express high levels of IL-17A and IL-17F, assigning them to a “Th17 biased” class. However, IL-27 potently downregulated IL-22 expression. To examine IL-22^+^ T cell pathogenicity, *in vitro*-generated cells were transferred into Rag1^−/−^ mice, retaining the modest reporter expression and inducing moderate colitis. In contrast, IL-22 expressers from colitic mice, transferred into secondary hosts, lost reporter expression, acquired high T-bet and modest IFNγ and IL-17 expression, and induced severe colitis. These findings are consistent with a model of strong polarization under optimal *in vitro* conditions, but a plastic state of T cells *in vivo*.

## Introduction

IL-22 was initially discovered as an IL-9- or activation-induced transcript in T cells (1). IL-22 performs important roles in host defense through its action on epithelial cells eliciting innate immune reactions [reviewed in Ref. (2, 3)]. In contrast, roles of IL-22 in pathology of bowel (4, 5), liver (6), and skin (7) have also been reported.

A number of sources of IL-22 have been described. Among T cells [reviewed in Ref. (2)], a human subset designated “Th22” has been distinguished from Th17 cells and is a major IL-22 producer (8, 9). This pattern in humans differs from the mouse, in which Th17 cells were reported to be major producers (7, 10). In man, Th1 cells were also shown to be capable of expressing IL-22 (11). In the mouse, expression of IL-22 has also been reported inTh1 cells (12), NKT cells (13, 14), and γδ T cells (15).

Several innate lymphocyte (ILC) subsets are reported to be IL-22 producers. These include NK cells in humans (16, 17) and mice (18, 19). Group 3 ILCs [reviewed in Ref. (20, 21)] consist of several subsets, most of them, including LTi cells (22, 23), are reported to be capable of producing IL-22. IL-22 production from other Group 3 ILC includes cells that express natural cytotoxicity receptors (NCR) (16, 18, 24–27) and NCR-negative ILCs (28, 29).

Transcription factors regulating murine IL-22 production include Stat3 that appears to be essential for expression in T cells (30, 31). Batf also appears to be required for expression and directly binds the IL-22 promoter (32). RORγt promotes expression, perhaps indirectly via upregulation of other receptors [reviewed in Ref. (2)]. Stimulation of the aryl hydrocarbon receptor (AHR) also promotes expression, but does not appear to be essential [reviewed in Ref. (2)]. The Notch pathway promotes IL-22 expression through what is believed to be an indirect mechanism (33). Lastly, c-Maf is a transcriptional inhibitor of IL-22, acting downstream of TGFβ (34), and possibly mediates the IL-27 inhibitory effect (35).

IL-22 expression in murine T cells [reviewed in Ref. (2)] is induced by IL-23 or IL-6 and is inhibited by TGFβ (7). IL-22 can be strongly stimulated by the combination of IL-23, IL-6, and IL-1 (36) and by IL-21 (30). Expression is promoted by ligands of the AHR, such as 6-formylindolo(3,2-b)carbazole (FICZ) (37) combined with αIFNγ and αIL4 (38). Most of these studies have examined induction of IL-22 expression *in vitro*, whereas *in vivo*, it is less clear what factors positively and negatively regulate its expression, as well as the characteristics of the IL-22-expressing T cells. To examine IL-22 expression *in vivo* and to characterize IL-22-expressing T cells, an IL-22 reporter mouse would advance our understanding of these cells.

In the current study, we describe a novel IL-22 reporter mouse. This was developed to address several questions. What cells express IL-22 under homeostatic conditions and during immune and inflammatory responses? Do T cells expressing IL-22 represent a stable lineage pattern, or are they plastic and capable of responding to a different cytokine milieu? Because IL-22 has both protective and pathogenic properties, are IL-22-expressing T cells protective or pathogenic? Using the reporter, we conclude that the major IL-22 expressers in gut are ILC3s and CD4 T cells. CD4 T cells expressing IL-22 showed greater stability of IL-22 expression when optimally polarized *in vitro* compared to those from an inflammatory site *in vivo*. However, even optimally polarized T cells from *in vitro* cultures demonstrated considerable plasticity after transfer *in vivo*. Finally, IL-22-expressing T cells, transferred *in vivo*, demonstrated marked pathogenicity in gut tissue, accompanied by loss of IL-22 expression and gain of expression of other cytokines, such as IFNγ and IL-17A.

## Materials and Methods

### Mice

C57BL/6 mice were purchased from the Animal Production Area, National Cancer Institute-FCRDC (Frederick, MD, USA). Rag1^−/−^ were originally purchased from The Jackson Laboratory (Bar Harbor, MN, USA) and maintained by homozygous breeding at NCI-Frederick. Three strains of IL-22-tdTomato mice have been produced at NCI, Frederick, MD, USA, and homozygous strains have been selected and maintained at the same animal facility. All mice used were 8–12 weeks old. NCI-Frederick is accredited by AAALAC International and follows the Public Health Service Policy for the Care and Use of Laboratory Animals. Animal care was provided in accordance with the procedures outlined in the Guide for Care and Use of Laboratory Animals (National Research Council; 1996; National Academy Press; Washington, DC, USA).

### Flow Cytometry and Antibodies

To perform surface staining, 1 × 10^6^ cells were placed in individual wells of a 96-well round bottom plate and incubated with the appropriate antibody cocktails for 15 min at 4°C on a slow rocker. After the staining, cells were fixed in a solution of 2% ultrapure formaldehyde (Polysciences, Inc., Warrington, PA, USA) in FACS buffer for 20 min on ice, washed twice, and analyzed the following day on the Canto II (BD Biosciences) or FACSCalibur (BD Biosciences). Intracellular staining was performed using Cytofix/Cytoperm Fixation/Permeabilization Solution Kit with BD GolgiStop (BD biosciences) according to the manufacturer’s instruction. Flow cytometry acquisition was performed on an LSRIISorp. Data were analyzed using FACS Express or FlowJo software (Tree Star, Inc., Ashland, OR, USA). Antibodies against CD45 (clone 30-F11, BD Pharmingen), CD3 (clone 145-2C11, BD Pharmingen), CD4 (clone GK1.5, BD Pharmingen), CD8 (clone 5H10, Biolegend), T-bet (clone eBio4B10, eBioscience), IL-17A (clone ebio17B7, eBioscience), IL-4 (clone B11B, Biolegend), IFNγ (clone XMG 1.2, eBioscience), IL-22 (clone A3.6M, eBioscience and clone poly5164, Biolegend), TGF-β (clone 11A5, Biolegend), IL-17F (clone ebio18F10, eBioscience), NKP46 (clone 29A 1.4, Biolegend), c-Kit (clone 2B8, Biolegend), Sca-1 (clone D7, BD Pharmingen), and CD127 (clone A7R34, eBioscience) were used.

### *In Vitro* T Cell Differentiation

Purified CD4 T cells from mouse spleen cells were performed by Dynal^®^ Mouse CD4 Cell Negative Isolation Kit (Life Technology) and cultured under Th22 conditions, including 1 μg/ml plate bound anti-CD3 (eBioscience), 0.5 μg/ml anti-CD28 (eBioscience), 10 μg/ml anti-IL4 (Biolegend), 10 μg/ml anti-IFNγ (Biolegend), 10 ng/ml IL-6 (Peprotech), 1 ng/ml TGF-β (Peprotech), and 200 nM 6-formylindolo(3,2-b)carbazole (FICZ, Sigma-Aldrich) for 4 days. Cells were harvested and sorted for tdTomato signal by flow cytometry using a FACSAria and cultured under different Th1, Th2, Th17, and Th22 conditions. For Th1 and Th2 condition, cells were stimulated with 1 μg/ml plate bound anti-CD3, 0.5 μg/ml anti-CD28 in the presence of 10 μg/ml anti-IL4 (Th1), 10 ng/ml IL-12 (Peprotech, Th1), 10 μg/ml anti-IFNγ (Th2), 10 μg/ml anti-IL12 (Biolegend, Th2), and 30 ng/ml IL-4 (Peprotech, Th2). For Th17 cell differentiation, cells were cultured with 1 μg/ml anti-CD3, 0.5 μg/ml anti-CD28, 10 μg/ml anti-IL4, 10 μg/ml anti-IFNγ, 10 ng/ml IL-6, 50 ng/ml IL-23 (R&D Biosystem), and 1 ng/ml TGF-β (Peprotech). Three days after activation, cells were restimulated with 500 ng/ml ionomycin and 50 ng/ml phorbol 12-myristate 13-acetate (Sigma-Aldrich) in the presence of GolgiStop for 5 h, after which IFNγ, IL-4, IL-17A, and IL-17F-producing cells were analyzed using a BDCytoFix/CytoPerm intracellular staining kit (BD Biosciences) following the manufacturer’s instructions.

### *In Vitro*-Generated Th22 Cell Transfer

Purified CD4 T cells cultured under Th22 polarization condition for 4 days, followed by sorting of tdTomato positive and tdTomato negative cells using a FACSAria (BD Biosciences). Sorted cells and CD4 T cells (cultured under neutralized condition for 4 days) were injected into the recipient mice (0.5 × 10^6^ cells/mouse). Mice were monitored twice a week with body weight, stool consistency, and occult/gross blood in stool using Hemoccult Slides (Beckman Coulter, Fullerton, CA, USA). Tissues were harvested 1 month following cell transfer.

### *In Vivo*-Generated Th22 Cell Transfer

Mesenteric lymph nodes were harvested from pre-colitis mice (4 weeks after CD4^+^CD45RB^high^ cells were transferred into Rag1^−/−^ mice, see Supplementary Material). IL-22 producing cells or non-producing cells were sorted of tdTomato fluorescent protein using FACSAria, followed by injection into second hosts (Rag1^−/−^ recipients, 0.5 × 10^6^ cells/mouse). Mice were monitored twice a week as described above, and tissues were harvested 1 month following cell transfer.

### RNA Extraction from Th22 Cells and RT-PCR

CD4 T cells were cultured under neutral (anti-CD3, anti-CD28) condition or Th22 conditions (anti-CD3, anti-CD28, anti-IL4, anti-IFNγ, 10 ng/ml IL-6, 1 ng/ml TGF-β, and FICZ) for 4 days, and sorted Th22 cells as described above were placed under Th1, Th2, Th17, and Th22 conditions for 3 days, followed by isolation of DNA-free total RNA using RNA II kit (MN) according to the provided protocol. Concentration and purity of RNA yield were established by spectrophotometry (Nanodrop, NanoDrop Technologies, Wilmington, DE, USA) and quality confirmed by electrophoresis. One microgram aliquots of each total RNA stock were converted into cDNA via hex primed reverse transcription (Thermoscript RT kit, Invitrogen). Th22 transcripts were then analyzed for relative amounts of Il22, tdTomato, and 18s ribosomal RNA via Taqman Gene expression analyses (Applied Biosystems) using an ABI7300 thermocycler. *C*_t_ values generated from each sample with the 18s-specific probe set were used to normalize expression of the two target genes (Il22 and tdTomato) using a Δ*C*_t_ method with correction for variation in amplification efficiency. Inflammatory cytokines and transcription factors were analyzed by semiquantitative RT-PCR using oligonucleotide primers (Integrated DNA Technologies; Coralville, IA, USA), as described previously (39). Briefly, the amplified PCR fragments were separated by electrophoresis on 1.2% agarose gel and visualized using SYBR Safe DNA gel stain (Invitrogen). To quantify the transcription levels, the amount of mRNA expression were normalized relative to the expression of HPRT mRNA using densitometric analysis by ImageJ 1.41 software.

### Gene Profile Analysis of Th22 Cells Treated with IL-27

*In vitro*-generated Th22-tdTomato cells were cultured with or without mIL-27 (20 ng/ml, Cytokine) for 4 days. Following the ionomycin and PMA stimulation with the GolgiStop for 5 h, cells were harvested and proceeded with total RNA preparation. RNA was then transcribed (Reverse Transcription kit, Qiagen) and analyzed by either RT-PCR or micro array method using RT2 Profiler PCR array – Th17 response array (PAMM-073Z plate, Qiagen) according to manufactures’ protocol.

### Histological Analysis and Scoring

Tissues (mesenteric lymph nodes, small and large intestine) from mice were fixed in 4% PFA overnight, replaced with 18% sucrose for 16–24 h, and then frozen for sectioned, and stained with either anti-RFP (abCam) or hematoxylin and eosin. H&E tissue sections were evaluated and graded in coded fashion by a veterinary pathologist (Miriam Anver). Semi-quantitative scale from 0 to 4 was used where histopathological changes were identified as minimal = 1, mild = 2, moderate = 3, and severe = 4. For the colon, cumulative histopathology scores were calculated based on the sum of individual changes of parameters (crypt hyperplasia, goblet cell depletion, lymphocytic infiltrates, eosinophils, neutrophils, gut intraepithelial neoplasm, crypt abscess, and chronic active inflammation). For the small intestine, cumulative histopathology scores were calculated based on the sum of individual changes of parameters (crypt hyperplasia, crypt loss, lymphocytic infiltrates, and chronic active inflammation).

### Statistics

Statistical analysis was performed using the GraphPad Prism 6.0 software. Data are expressed as mean ± SEM. The Student two-tailed unpaired, parametric *t* test was used to assess statistical differences between two groups. Asterisks indicate statistical differences, *p < 0.05, **p < 0.01, and ***p < 0.001.

## Results

### IL-22 Reporter Construction and Transgene Expression

**Figure 1:**
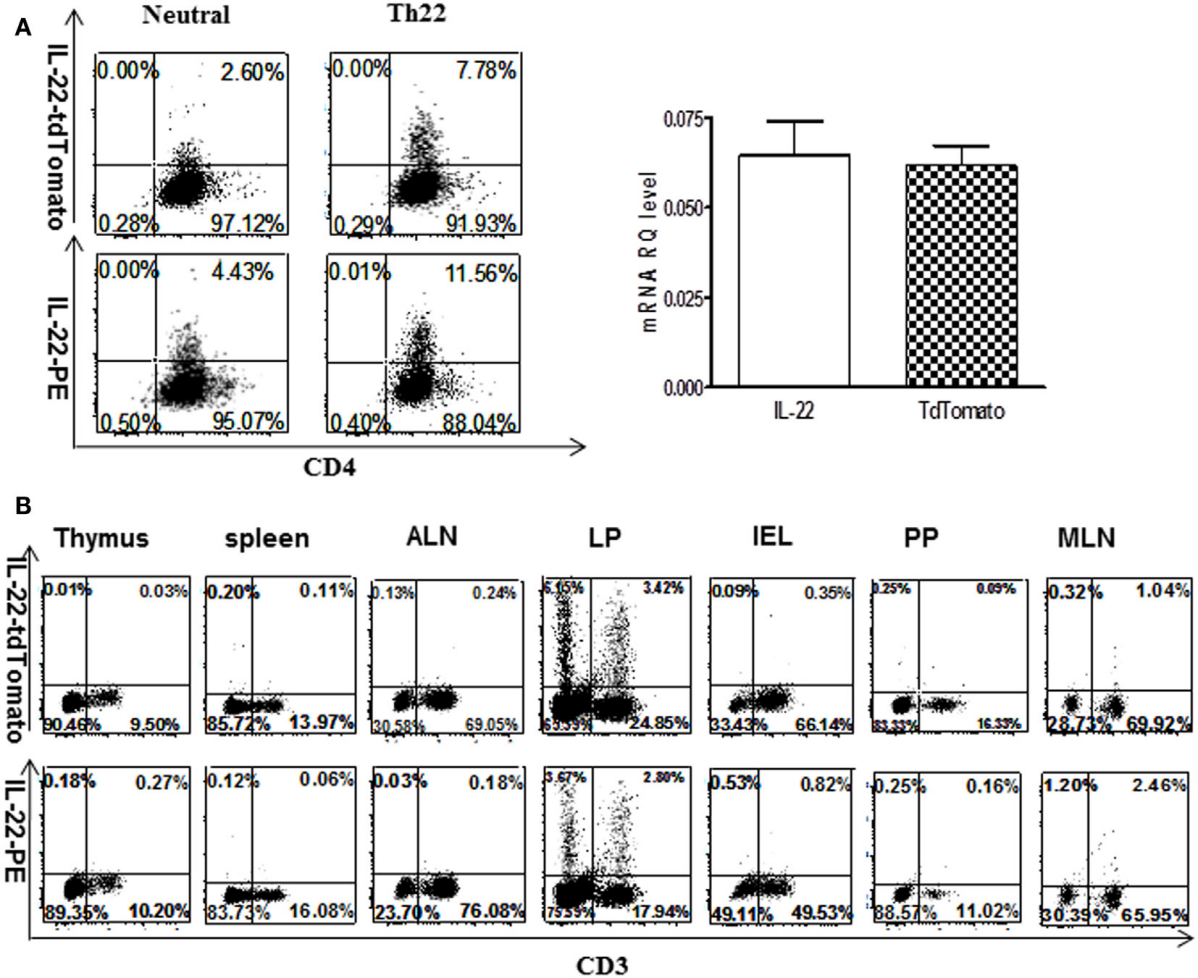
**Correspondence of IL-22 reporter expression to endogenous IL-22**. **(A)** Spleen CD4^+^ T cells from reporter mice were cultured 4 days in the presence of IL-22-inducing conditions (FICZ + IL-6 + anti-IL-4 + anti-IFNγ + IL-23). Cells were treated with GolgiStop for 5 h and stained for intracellular IL-22 or analyzed for the tdTomato reporter by real-time RT-PCR (data are expressed as mean ± SEM). Numbers in quadrants indicate percent of CD3^+^ cells. Data indicate three independent experiments. **(B)** *In vivo* expression of IL-22 or reporter in different tissues. Cells were examined for the tdTomato reporter or stained with anti-IL-22. MLN, mesenteric lymph node; ALN, axillary lymph node; PP, Peyers patch; IEL, intraepithelial cells isolated from small intestine; LP, lamina propria cells purified from small intestine. Numbers in quadrants indicate percent of lymphocyte cells. Data are representative of two or more experiments.

A murine IL-22 reporter transgene was created using recombineering to modify a bacterial artificial chromosome, as previously described (40), introducing tdTomato into exon 1 (Figure S1 in Supplementary Material). Because the signal sequence was disrupted by design, this results in accumulation of the reporter in expressing cells, enabling their detection and isolation by flow cytometry. The transgene was introduced into C57Bl/6 mice by standard methods, and several founder lines were bred to homozygosity. The selected founder line showed fidelity of expression to the endogenous IL-22 gene by several criteria as follows. *In vitro*, reporter expression was induced in CD4 T cells by the same combination of stimuli as the IL-22 gene (Figure 1A), and there was a similar quantitative expression of reporter transcripts compared to the endogenous IL-22 gene measured by quantitative RT-PCR. *In vivo*, the same lymphoid subsets expressed the reporter and IL-22 (Figure 1B) mostly in lamina propria (LP) cells from gut, but not other gut-associated lymphoid tissue (GALT), axillary lymph node (ALN), spleen, or thymus.

### Phenotype of Lymphoid Cells Expressing the IL-22 Reporter

**Figure 2:**
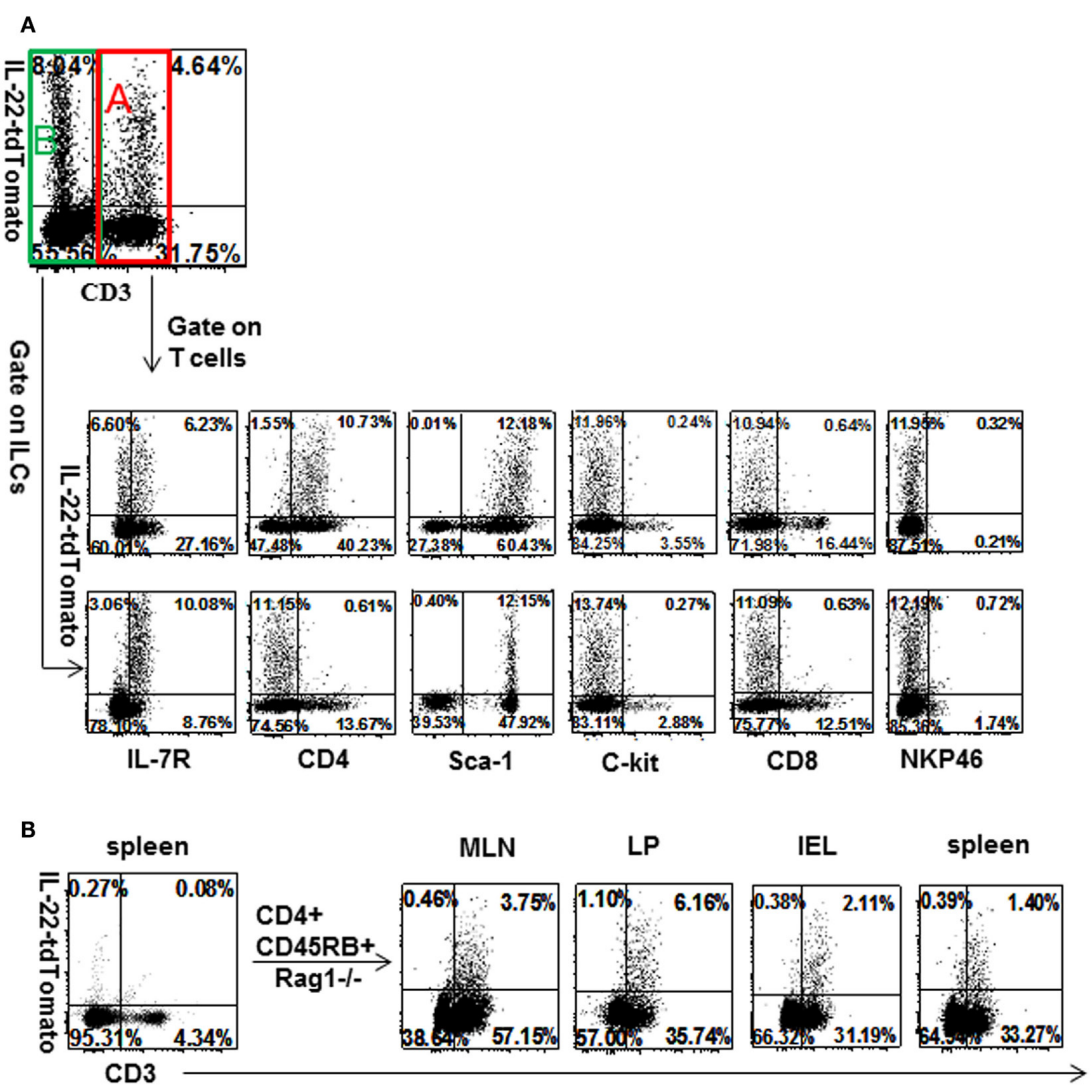
**Identification of IL-22-producing T cells and innate lymphoid cells in normal LP and inflamed GALT**. **(A)** Lymphoid cells from LP of unmanipulated IL-22 reporter mice were stained for different markers of innate lymphocytes and T cells. **(B)** CD4^+^CD45Rb^hi^ T cells of reporter mice were transferred into Rag1^−/−^ mice and lymphoid cells from gut-associated lymphoid tissue were examined 4 weeks later (*n* = 4, data representative of three independent experiments). Numbers in quadrants indicate percent of CD45^+^ cells.

Lymphoid cells from LP were analyzed for markers on cells expressing the IL-22 reporter under homeostatic conditions. The more frequent population was ILC3, identified by the criteria that they expressed IL-7R and lacked CD3 (Figure 2A). The less frequent population was CD4 T cells (Figure 2A; Figure S2 in Supplementary Material). Both populations expressed Sca1, neither expressed cKit, NKp46, or CD8. To examine inflamed tissue, colitis was induced by transfer of reporter CD4^+^CD45Rb^hi^ T cells into Rag1^−/−^ mice. The initially transferred T cell population from spleen contained very few reporter-positive cells (Figure 2B). Four weeks after transfer, mice were precolitic and showed an expansion of IL-22 reporter T cells in MLN, LP, IEL, and spleen (Figure 2B). At the onset of colitis, 8 weeks after T cell transfer, the tdTomato signal diminished in mesenteric lymph nodes (declining by 6 weeks as seen in Figures S3A,B in Supplementary Material), indicating that IL-22 itself was not directly associated with gut pathology, as will be discussed in a later section.

### IL-22-Expressing T Cells: Stronger Polarization *In Vitro* than *In Vivo*

**Figure 3:**
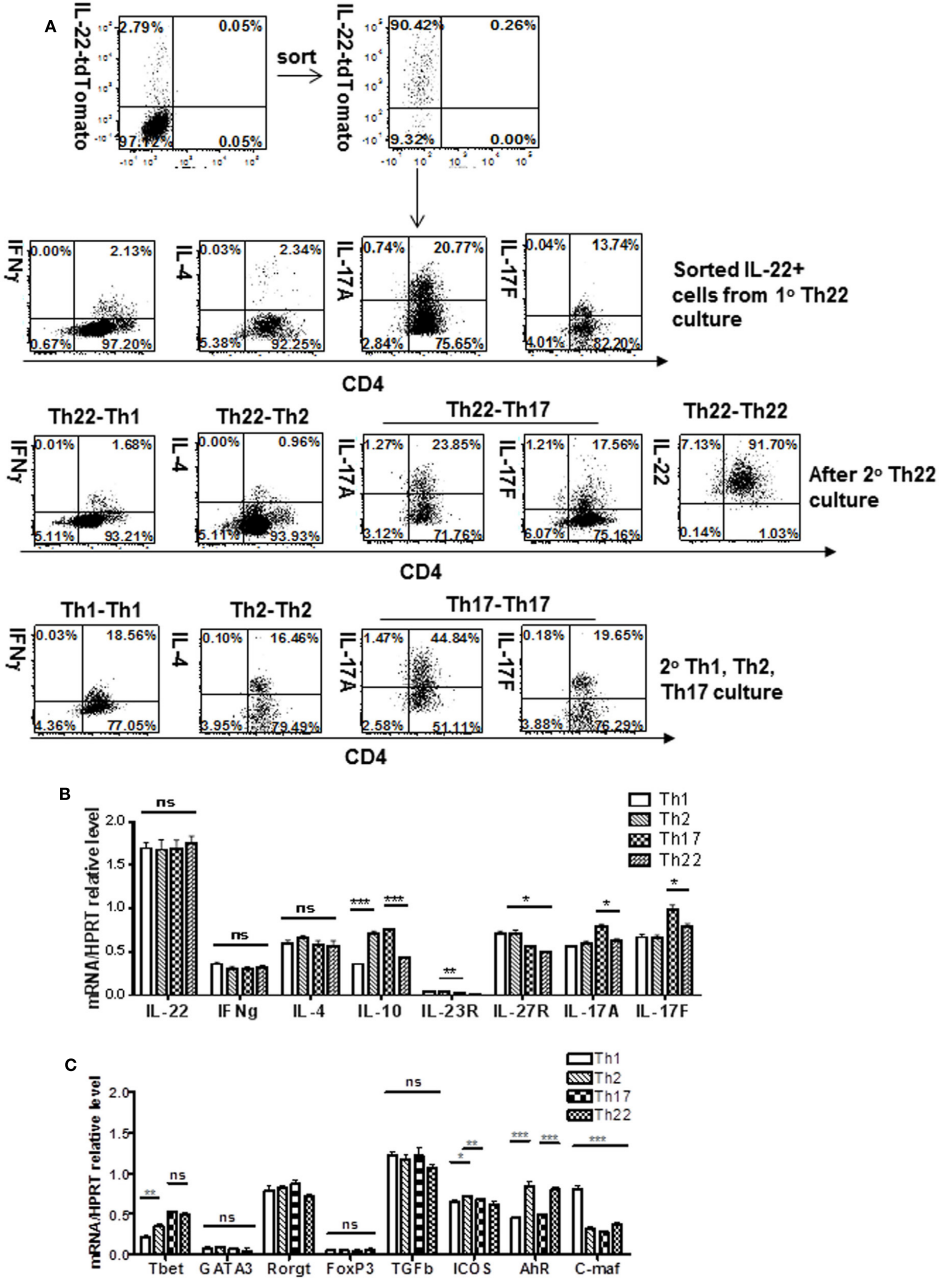
**IL-22-producing T cells generated *in vitro*: stability in 2° Th1, Th2, or Th17 culture**. T cells from IL-22 reporter mice or non-reporter WT mice were generated in a 1° culture as shown in Figure 1A and sorted for reporter expression. Th1, Th2, or Th17 cells were generated in control 1° cultures. Sorted cells from 1° Th22 cultures (or control Th1, Th2, or Th17 cultures) were then placed in a 2° culture for 3 days under Th1 (αCD3, αCD28, αIL-4, IL-12), Th2 (αCD3, αCD28, αIFNγ, IL-4), or Th17 (αCD3, αCD28, αIL-4, αIFNγ, IL-6, IL-23, TGF-β, and IL-1β) conditions. **(A)** Cells were treated with GolgiStop and stained for IFNγ, IL-4, or IL-17, and analyzed for IL-22 reporter expression. Numbers in quadrants indicate percent of CD45^+^ cells. **(B)** Cells were analyzed for expression of cytokines. Data represent mean ± SEM (*n* = 6 total samples cumulative from two separate experiments), *p < 0.05, **p < 0.01, and ***p ≤ 0.001; ns, not significant, determined by *t*-test. **(C)** Cells were analyzed for expression of what were formerly called “master regulators” T-bet, Gata3, RORγt, FoxP3, or the membrane protein ICOS or transcription factors AHR or cMaf, an inhibitor of IL-22 expression. Gene expression was normalized to Hprt levels. Data are expressed as mean ± SEM (*n* = 8 total samples cumulative from two separate experiments), *p < 0.05, **p < 0.01, and ***p ≤ 0.001; ns, not significant, determined by *t*-test. Data represent two independent experiments.

To evaluate plasticity of the IL-22 lineage when generated *in vitro*, CD4 T cells were first cultured 4 days under IL-22 conditions (αCD3 + αCD28 + FICZ + IL-6 + IL-23 + αIL-4 + αIFNγ). Expressing cells were then enriched by sorting and placed in secondary cultures for 3 days under conditions favoring differentiation into other CD4 subsets, such as Th1, Th2, or Th17. Three days of Th1 culture conditions neither extinguished IL-22 reporter expression nor induced expression of the Th1 signature cytokine IFNγ (Figure 3A) compared to positive controls (Figure 3A; Figure S4 in Supplementary Material). Th2 culture conditions similarly failed to extinguish IL-22 reporter expression or induce expression of the Th2 signature cytokine IL-4. On the other hand, IL-17 was coexpressed in about 15% of cells following the initial “IL-22” culture and increased under subsequent Th17 culture conditions, although some IL-17 expressers extinguished IL-22 reporter expression. Other cytokines and receptors were examined at the level of transcripts (Figure 3B), the only one showing major modulation was IL-10, which was upregulated under Th2 or Th17 conditions. These results indicate that T cells induced *in vitro* to express IL-22 were strongly polarized away from the Th1 or Th2 lineages. On the other hand, the relationship with Th17 expression is consistent with a single lineage capable of both IL-22 and IL-17 expression depending on environmental signals. We will use the term “Th22” merely as a convenient term to describe T cells currently expressing the IL-22 reporter.

Having examined the plasticity of T cells expressing IL-22 under different conditions, these cells were examined further for expression of, what had been termed “master regulators,” transcription factors known to control genes of the major subsets (Figure 3C; Figures S5 and S6 in Supplementary Material). The master regulators for Th1, Th2, Th17, and Tregs are T-bet, Gata3, RORγt, and FoxP3, respectively. RORγt, high under IL-22 conditions, remained relatively stable under subsequent Th1, Th2, or Th17 conditions, perhaps contributing to the relative stability of IL-22 expression. We noted that RORγt was not uniquely high in Th22 cells but was also high in normal spleen or Th17 cells (Figure S6 in Supplementary Material). Gata3 expression was extremely low in IL-22-expressing T cells and not increased in subsequent Th2 conditions, accounting in part for the absence of IL-4 expression. On the other hand, T-bet was relatively well expressed together with IL-22 in the first and subsequent cultures, but was not accompanied by IFNγ expression (Figures 3A,B), indicating it is insufficient as a “master regulator.” FoxP3, required for Tregs, was extremely low in Th22 cells. High TGFβ expression was observed under all conditions, including normal spleen (Figure S6 in Supplementary Material), and thus was not unique to Th22 cells. Comparison with conventional Th1, Th2, and Th17 cells (Figure 5A in Supplementary Material) shows that in cells from conventional Th1, Th2, and Th17 cultures, their signature cytokines, IFNγ, IL-4, and IL-17A/F, are higher than in cells derived from Th22 cultures recultured under the same conditions. Thus, the Th22 bias generated from optimal cultures was relatively stable in secondary cultures optimal for other subsets.

Several transcription factors that are thought to regulate IL-22 expression were evaluated for plasticity, including the positive regulator and AHR, and the negative regulator c-Maf (Figure 3C). AHR was strongly expressed in Th22 cells and somewhat suppressed under subsequent Th1 and Th17 conditions, and yet, despite its usual positive association with IL-22, was not accompanied by downregulation of IL-22. c-Maf was expressed in Th22 cells and strongly increased by Th1 conditions, but despite its association with inhibiting IL-22 expression was not accompanied by downregulation of IL-22. Expression of the membrane protein ICOS, which has been associated with IL-22 expression, was strongly expressed in Th22 cells, but little affected by subsequent culture conditions. Comparing recultured Th22 cells to cells from primary cultures favoring Th1, Th2, or Th17 (Figure S5B in Supplementary Material) showed the latter transcribed much higher levels of signature regulators including T-bet, GATA3, RORγt, and FoxP3.

**Figure 4:**
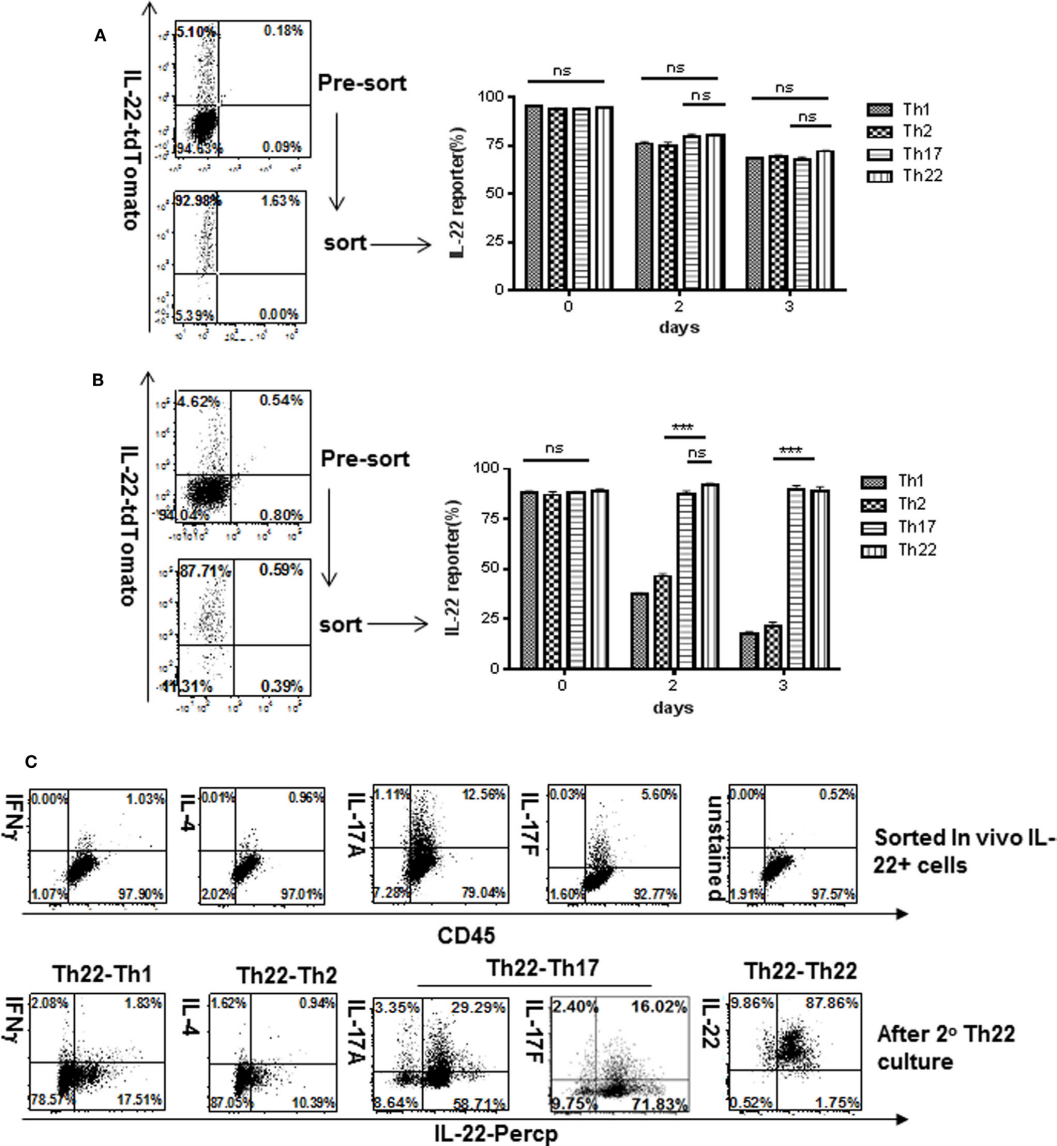
**Stability of IL-22 production comparing T cells generated *in vitro* versus *in vivo***. T cells expressing the IL-22 reporter were generated **(A)** *in vitro* for 4 days under conditions as shown in Figure 1 or **(B,C)** *in vivo* in pre-colitic mice as shown in Figure 2, at 4 weeks. Cells were sorted for reporter expression, placed in Th1, Th2, or Th17 culture conditions (as shown in Figure 3) for various times then analyzed for reporter expression. Numbers in quadrants indicate percent of CD4^+^ cells **(A,B)**, and lymphocyte cells **(C)**. Bar graph represents percentage of CD4 T cells (*n* = 3), and plotted as mean ± SEM. *p < 0.05 and ***p ≤ 0.001; ns, not significant, determined by *t*-test. Data are indicative of three independent experiments.

Having analyzed plasticity of IL-22 expressers generated *in vitro*, we next examined IL-22 expressers generated in a pathological setting *in vivo* by colitis induction. CD4 T cells from reporter mice were injected into Rag1^−/−^ hosts to induce colitis. Four weeks later, IL-22-reporter-expressing T cells were purified from MLN and placed into Th1, Th2, or Th17 culture conditions. Under Th1 or Th2 conditions, these cells showed considerably less stability of reporter expression (Figures 4B,C; Figure S8 in Supplementary Material) than did IL-22 expressers generated *in vitro* (Figure 4A) and acquired modest expression of Th1 or Th2 cytokines. This suggests that T cells, under physiological or pathological conditions, may retain much more plasticity than suggested by optimal priming *in vitro*.

### Pathogenicity of Th22 Cells

IL-22, under different conditions has been reported to promote epithelial repair, or on the other hand, to promote inflammation. To evaluate the pathogenicity of *in vitro*-generated Th22 cells, a colitis model was used.

#### *In Vitro*-Generated Th22

**Figure 5:**
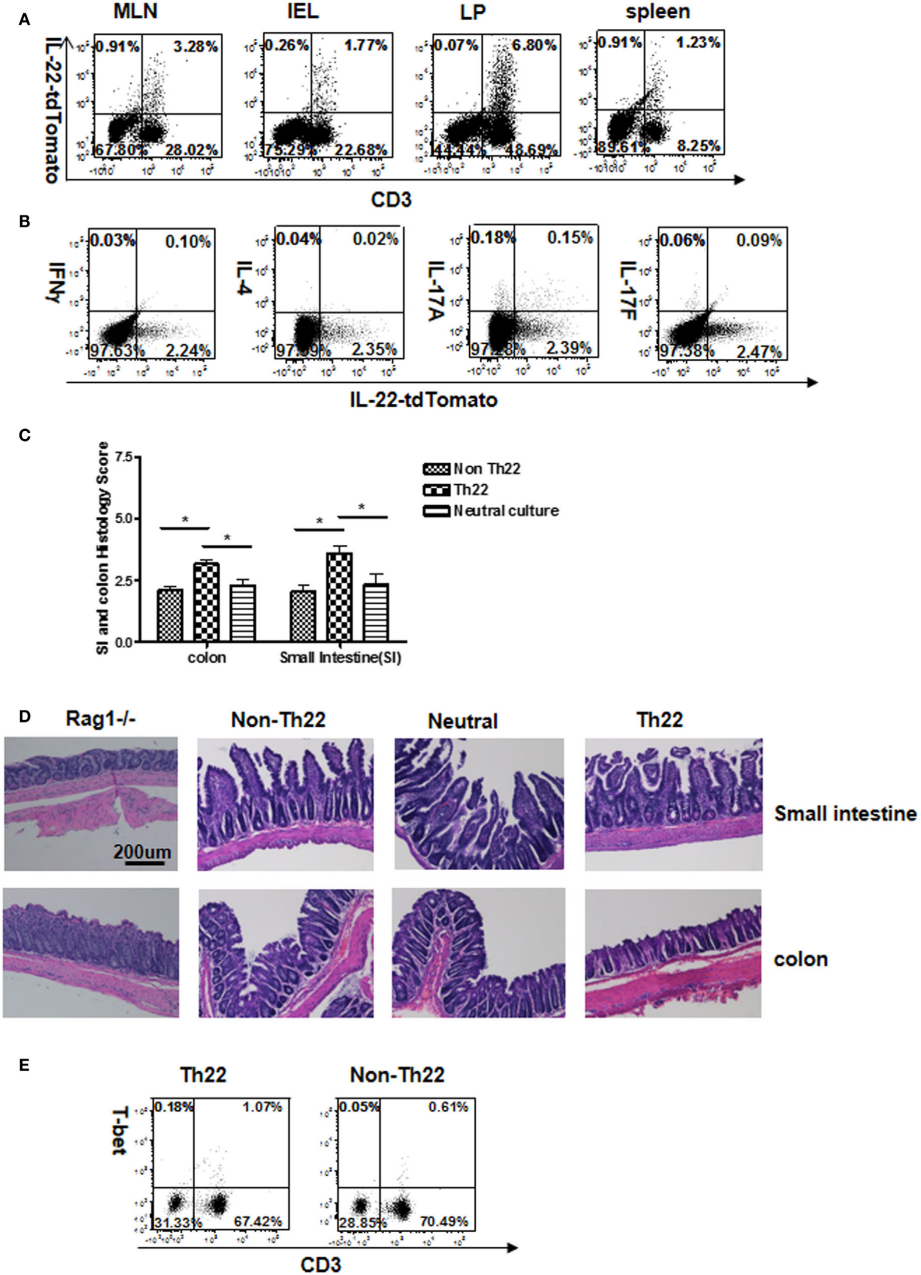
**Stability of IL-22 production and pathogenicity by *in vitro*-generated Th22 cells transferred *in vivo***. T cells expressing the IL-22 reporter were generated *in vitro* for 4 days. Cells were sorted for reporter expression and mice received cells from a “neutral culture” (αIFNγ + αIL-4 + αCD3 + αCD28) versus sorted IL-22 (+) or (−) cells from a Th22 culture as shown in Figure 1. Four weeks after receiving reporter-positive cells and inducing colitis **(A)**, T cells from GALT were analyzed for reporter expression. Numbers in quadrants indicate percent of CD45^+^ cells. Data are representative of two experiments. **(B)** Different cytokines were determined in LP cells by intracellular staining and flow cytometry. Numbers in quadrants indicate percent of CD45^+^ cells. Note: comparing **(A,B)**, tdTomato fluorescence was somewhat reduced by fixation and permeabilization in **(B)**. Data are representative of two experiments. **(C)** Intestinal tissues were evaluated histologically for ileitis and colitis. Histopathological scores were determined for the distal colon and distal small intestine (*n* = 8 total mice per group cumulative from two separate experiments). **(D)** H&E staining for representative sections in which “Rag1^−/−^” shows control sections from mice that received no T cells. **(E)** Flow cytometry analysis of T-bet in lamina propria cells from Rag1^−/−^ mice receiving Th22 or non-Th22 cells. Numbers in quadrants indicate percentage of CD45^+^ cells. Data are representative of two experiments.

T cells were cultured under IL-22-promoting conditions, purified for IL-22 reporter expression, and transferred into Rag1^−/−^ recipients to generate colitis. Four weeks later, analysis of GALT showed that a small fraction of transferred CD3^+^ cells had retained reporter expression (Figures 5A,B) or IL-22 transcripts (Figure S8 in Supplementary Material). A very small fraction expressed IL-17A, and none expressed IFNγ or IL-4. T cells derived from IL-22 expressers induced an inflammatory response in both small and large bowel, which was significantly stronger than that induced by IL-22 negative cells from the same culture, or CD4 T cells from “neutral cultures” (Figures 5C,D). By “neutral culture,” we refer to polyclonally stimulated T cells, but without other culture conditions favoring a particular subset. Thus, we blocked IFNγ and IL-4, and did not add cytokines favoring differentiation into different subsets. This seemed like a reasonable control for Th22 culture conditions, and the transferred “neutral” cells showed less pathogenicity compared to sorted Th22 cells. Note that T-bet was not detected in the mice receiving Th22 cells generated *in vitro* (Figure 5E); this may have relevance by comparison to *in vivo*-generated Th22 as will be shown.

#### *In Vivo*-Generated Th22

**Figure 6:**
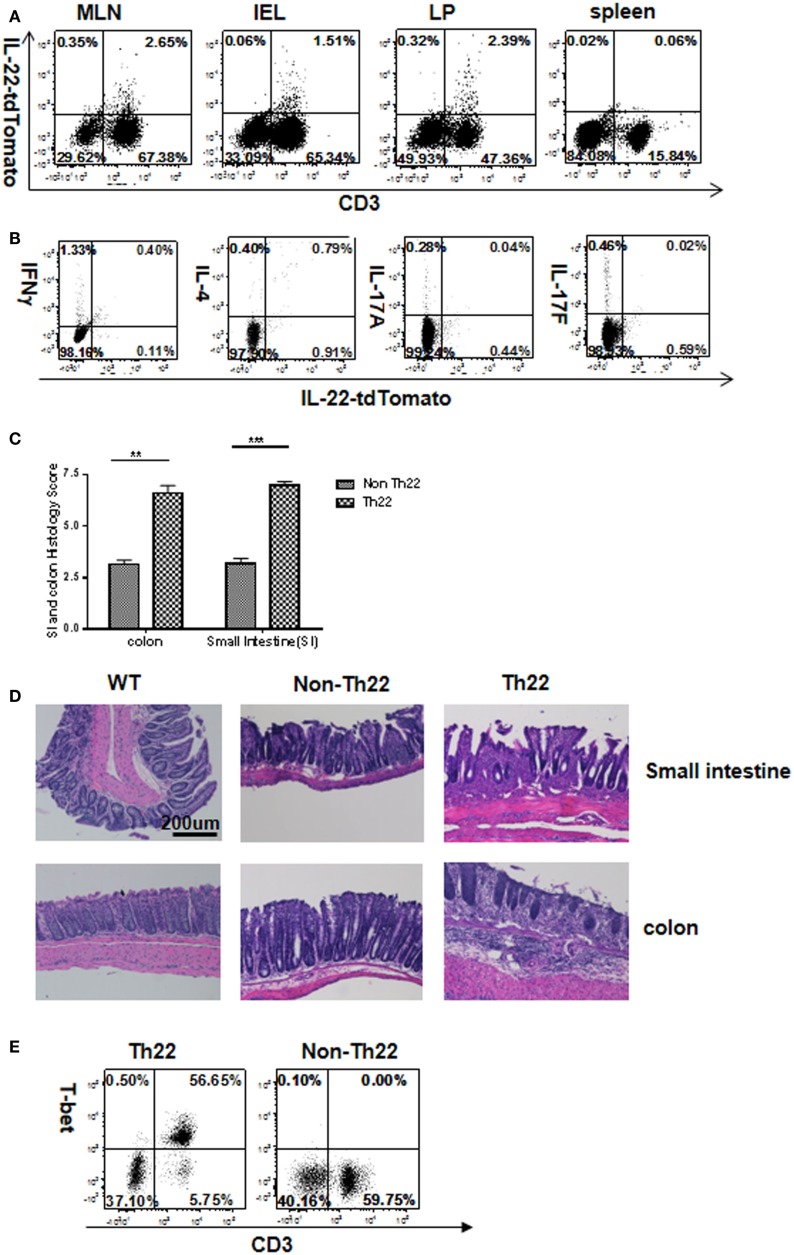
**Instability of IL-22 production and high pathogenicity of *in vivo*-generated Th22 cells transferred into secondary hosts**. **(A)** T cells from IL-22 reporter mice were first transferred into Rag1^−/−^ mice to induce colitis. Four weeks later, MLN T cells expressing the IL-22 reporter were transferred to secondary hosts. After 4 weeks in mice receiving IL-22 reporter-positive cells **(A)**, T cells from gut-associated lymphoid tissues were analyzed for reporter expression. Numbers in quadrants indicate percentage of CD45^+^ cells. Data are representative of two experiments. **(B)** Intracellular cytokines were examined in lamina propria cells by flow cytometry. Numbers in quadrants indicate percent of CD45^+^ cells. Data are representative of two experiments. **(C)** Intestinal tissues were evaluated histologically for inflammatory bowel disease. Histopathological scores were determined for the distal colon and distal small intestine (*n* = 8 total mice per group cumulative from two separate experiments). **(D)** H&E staining for representative sections. **(E)** Flow cytometry analysis of T-bet in lamina propria cells from *in vivo* transfer of T cells expressing or not expressing the IL-22 reporter. Numbers in quadrants indicate percentage of CD45^+^ cells. Data are representative of two experiments.

Since *in vitro*- and *in vivo*-generated Th22 cells differed in their plasticity, we compared their pathogenicity. Two successive T cell transfers (each of 4 weeks) were used to evaluate Th22 cells. In the first transfer, T cells from IL-22 reporter mice were transferred into Rag1^−/−^ mice to generate Th22 cells *in vivo*. Four weeks later at the precolitic stage, these *in vivo*-generated Th22 cells were purified from mesenteric lymph node and transferred into secondary Rag1^−/−^ recipients and analyzed 4 weeks later. Exceedingly, few of the transferred CD3^+^ cells retained reporter expression in GALT 4 weeks after secondary transfer (Figure 6A). Moreover, some of the transferred T cells had gained expression of IFNγ, IL-17A, and IL-17F, while losing IL-22 reporter expression (Figure 6B). This is consistent with preceding results showing that IL-22 expressers that are optimally polarized *in vitro* do not reflect the greater plasticity of T cells under physiological or pathological conditions that occur *in vivo*. On the other hand, colitis was much more strongly induced by *in vivo* than *in vitro* Th22 (Figures 6C,D; Figure S7 in Supplementary Material). Pathogenicity may reflect the modest switch to expression of the pathogenic cytokines IFNγ, IL-17A, and F (Figure 6B), although in other models of inflammatory bowel disease (IBD), the percentage of IFNγ producers can be much higher (41). The decrease in IL-22 reporter expression could also account for increased pathogenicity (compare Figures 6B and 5B) if IL-22 had a protective effect. The striking upregulation of T-bet (Figure 6E) may account for increased IFNγ or other pathogenic features.

### Effect of IL-27 on Th22 Cells

**Figure 7:**
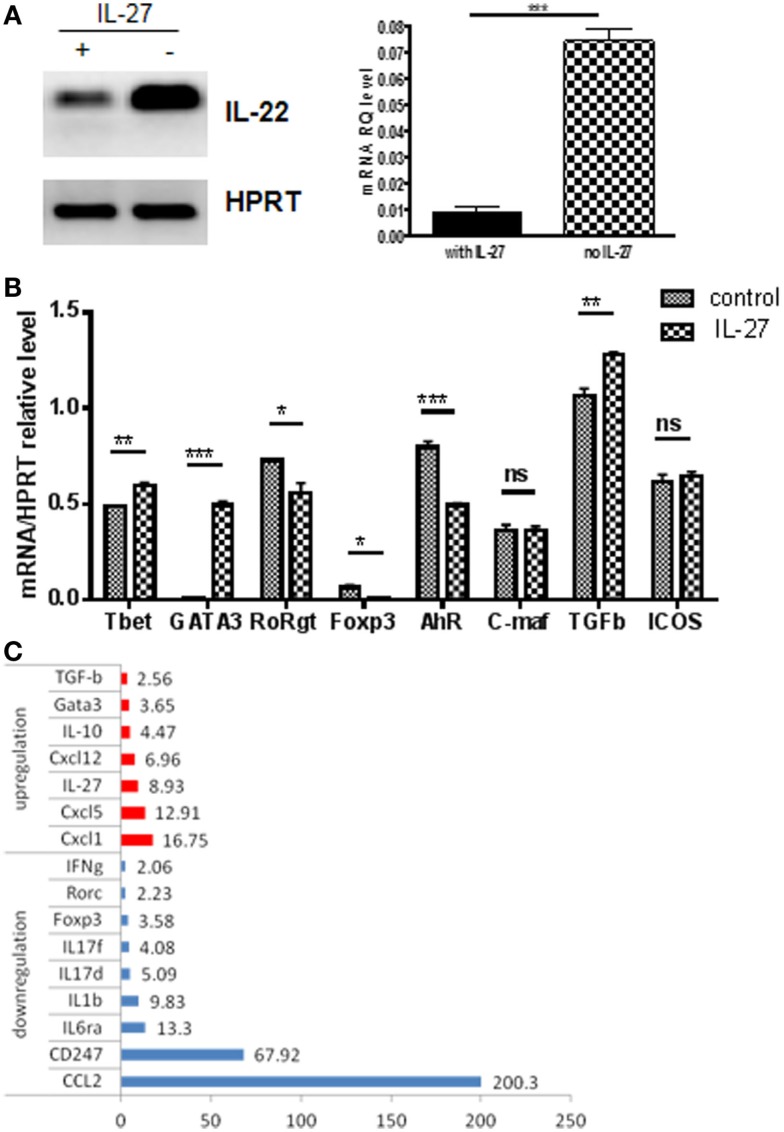
**IL-27 effects on products of IL-22 expressers**. T cells expressing the IL-22 reporter were generated *in vitro* as shown in Figure 1. Cells were sorted for reporter expression and treated with IL-27 under conditions favoring IL-22 expression or other Th differentiation conditions for 4 days and evaluated for transcripts by PCR. **(A)** IL-22 expression. Data are analyzed by regular PCR (left panel) or real-time PCR (right panel, mean ± SEM, *n* = 3). **(B)** Regulators and transcription factors were examined by qPCR. Gene expression was normalized to Hprt levels. Data are expressed as mean ± SEM (*n* = 3) *p < 0.05, **p < 0.01, and ***p ≤ 0.001; ns, not significant, determined by *t*-test. Data are representative of two independent experiments. **(C)** Cytokines from IL-27-treated Th22 cells were evaluated by microarray using Th17 response PCR array. Data represent the fold changes compared to non-treated cells. Data are representative of two independent experiments.

IL-22 expression by human T cells was recently reported to be suppressed by IL-27 (42). In the mouse, it has been shown that IL-27 can inhibit Th17 development (43, 44). IL-27 receptor transcripts were detected (Figure 3B), so we evaluated the effect of IL-27 on Th22 first generated *in vitro*. IL-27 strongly inhibited IL-22 expression (Figure 7A). This is consistent with observed reductions in RORγt and AHR and upregulation of TGFβ (Figure 7B). The cytokine profile shifted to a generally less inflammatory pattern showing reductions in IL-17s, Ccl2, Ccl5, and IL-9, and increased IL-10 and IL-27 itself (Figure 7C).

## Discussion

A novel IL-22 reporter mouse was developed, enabling us to purify expressing cells and examine their properties, including plasticity, lifespan, and pathogenicity. Under homeostatic conditions, IL-22 reporter expression was prominent in the GALTs in ILCs and CD4T cells. T cell expression was relatively stable if IL-22 expression was induced *in vitro*, but not *in vivo*. There was strong association with the capacity to express IL-17 and no coexpression with IFNγ or IL-4 during *in vitro* cultures that can promote expression of the latter. These findings are consistent with a single T cell lineage having the capacity to express either or both IL-22 and IL-17, which is distinct from Th1 and 2 lineages. IL-22-expressing T cells, generated *in vivo* or *in vitro*, induced IBD following transfer *in vivo*. Transferred T cells were shown to persist *in vivo* for at least 2 months, although losing IL-22 expression. Although loss of IL-22 expression could be explained by expansion of contaminating IL-22-reporter negative cells, cytokines are not constitutively expressed by T cells, so it is reasonable to interpret this as loss of IL-22 expression from a lineage previously expressing it.

The complex regulation of IL-22 expression in T cells has been the subject a very thorough review (2). There are many demonstrated regulators of IL-22 expression in these cells. Positive extracellular stimuli include IL-6, IL-23, IL-21, IL-1β, IL-18, and Notch ligands. TGFβ and the ICOS pathway are negative stimuli. Transcription factors include STAT3 and Batf, which are essential, AHR and RORγt, which augment expression, and c-Maf and IRF4, which inhibit. The relative stability of the mouse Th22 phenotype that was generated *in vitro* could relate to persistence of receptors or transcription factors. Phenotypic stability could also relate to epigenetic modifications of the IL-22 gene itself as has been discussed in the context of other cytokine genes in T cell subsets (45), and it will be interesting to analyze the IL-22 gene for such modifications.

A recent study employed a fate reporter for IL-22 (46), in which cells were permanently marked after activating the *IL-22* locus. As in our study, gut ILC3s were marked under homeostatic conditions. In the T cell lineage, perhaps because that reporter was slow to react, there was little homeostatic expression or *in vitro* induction. However, under inflammatory conditions, marked CD4 cells, as in our study, could express IFNγ, consistent with plasticity, rather than fidelity to a stable Th22 lineage.

Our findings contrast with some others regarding the expression of NKp46 on ILCs expressing IL-22. We observed background staining for NKp46 (Figure 2) (using two different antibodies), similar to that of Sonnenberg et al. (3, 29) p. 202. However, other studies have observed IL-22 expression from NKp46^+^ ILCs [reviewed in Ref. (25) p. 203]. These discrepancies may partly arise not only from the differences in animal colonies or isolation procedures but also from the frequent use of stimulants *ex vivo* (such as IL-23), whereas our observations are on cells directly isolated from mucosa.

Although early studies had concluded that *in vitro*-generated Th1 and Th2 cell types were not interconvertible (47, 48), more recent studies *in vivo* reveal, for example, Th2 reprograming to express Th1 features (49). Th17 cells were reported to be non-plastic in one study *in vivo* (50), whereas a number of studies *in vivo* reported plasticity (51–53). In our study of CD4 IL-22 expressers, reprograming appeared to be inversely correlated with intensity of polarization. “Strong” *in vitro* polarization yielded cells with relatively durable IL-22 expression under Th1 or 2 culture conditions. “Weaker” *in vivo* polarization in inflamed tissue yielded IL-22 expressers that lost expression under Th1 or 2 culture conditions. This variable range of polarization is reminiscent of Th1, Th2, and Th17 phenotypes which also become strongly polarized with optimal stimuli *in vitro* and were once thought to be stable lineages [reviewed in Ref. (45)]. However, T cells in an *in vivo* milieu, as in our study, appear less differentiated and have more stem-like properties (54), with the potential for a broad repertoire of responses regulated by the cytokine milieu at the moment. Even more strongly polarized IL-22 expressers generated *in vitro* lost expression when transferred into an inflammatory milieu *in vivo*. The loss of IL-22 expression that we observed *in vivo* could be due to several possible mechanisms. Loss of expression could be due to cytokines that suppress, such as TGFβ or IL-27 as shown here. Loss of expression could also be due to insufficient levels of inducing cytokines, such as IL-23 or IL-6, or to other unknown mechanisms.

Human Th22 cells exhibit less plasticity than mouse T cells expressing IL-22 and were reported to maintain IL-22 expression under Th1 or 2 culture conditions (55). Also, in contrast to IL-17 induction that was readily induced in mouse IL-22 expressers in our experiments, human Th22 cells were not amenable to IL-17 induction (55) (although some human Th17 coexpress IL-22). Thus, human “Th22” cells appear to constitute a subset distinct from Th17 cells (9), whereas mouse cells, from our data are “Th17 biased” (Figures 3A,B).

Although IL-22 expression could be extinguished under Th1 or Th2 culture conditions, we did not observe induction of Th1 or Th2 signature cytokines, IFNγ or IL-4, respectively, under *in vitro* conditions (Figures 3A,B). However, under inflammatory conditions, *in vivo*, IFNγ and IL-4 were induced from these cells (Figure 6), perhaps contributing to the pathogenicity of the transferred cells (Figure 6). In the mouse, primary T cells had been reported to express IL-22 transcripts after culture under Th1 (but not Th2) conditions (11); however, IL-22 protein was detected only under Th17, but not Th1 or 2 conditions (10). Human T cells have been reported to coexpress IFNγ (but not IL-4) with IL-22 (8, 9). Perhaps surprisingly, T-bet, the “master regulator” of IFNγ, was relatively well expressed together with IL-22 in the first and subsequent cultures, but was not associated with IFNγ expression, in contrast to other conditions reporting coexpression (36). This could be explained by a lack of accessibility of the IFNγ gene due to a failure of pioneer nuclear factors to render its opening (45). This illustrates the point that the term “master regulator” is overly simplistic as has been shown experimentally in several lineages (56, 57).

Pathogenicity of Th22 cells was manifested as severe IBD following transfer into Rag1^−/−^ mice. This pathogenicity was considerably greater if the Th22 cells derived from precolitic mice (Figure 6) than from Th22 cultures (Figure 5); however, the former would have TCR specificities for gut microbiota, whereas the latter would have random TCR specificities. Although IL-22 has been shown to contribute to pathogenicity in some models (6, 7, 28), pathogenicity of Th22 cells in our experiments seems unlikely to be due to IL-22 itself. Although our experiments do not directly address whether IL-22 itself is pathogenic, we suggest that it is not pathogenic because its expression does not correlate with disease by several criteria. First, the peak of IL-22 expression in colon and ileum (Figure S3A in Supplementary Material) at 4 weeks after transfer precedes the peak of pathology at 8 weeks. Second, the most pathogenic cells (Figure 6) express much less IL-22 than the less pathogenic cells (Figure 5). Third, there is a dramatic loss of expression after transfer, T cells decline from nearly 90% positive (Figure 4B) in the transferred population down to 1 or 2% in GALT (Figure 6A) – it therefore seems unlikely that the pathology is due to IL-22. Although these are correlations, they are more consistent with IL-22 *per se* being non-pathogenic, whereas the T cells that once expressed IL-22 are capable inducing pathology.

Since the reporter became silenced in most cells following transfer. IL-22 could have a protective role in that higher expression (Figure 5C) was associated with less pathology than low expression (Figure 6C). IL-17A and F would be candidates for pathogenicity since they are clearly pathogenic in this model and the cells were shown to produce modestly higher IL-17A and F as well as IFNγ and IL-4 (Figure 6), which can also be colitogenic. The higher colitogenic population was also associated with a striking increase in T-bet expression (Figure 6C). Silencing of IL-22 following transfer could result from a lack of inducing factors *in vivo*, or active suppression, for example, by IL-27 (Figure 7) or TGFβ (34). Future studies could address the mechanism of Th22 plasticity *in vivo*. Determining this mechanism could lead to methods for maintaining IL-22 expression in Th22 cells, and inhibiting IFNγ and IL-4, leading to a therapeutic benefit in IBD.

## Author Contributions

WS: designed and conducted experiments and wrote the paper, JH: conducted experiments, MM: conducted experiments, WL: designed and conducted experiments, and SD: directed the project and wrote the paper.

## Conflict of Interest Statement

The authors declare that the research was conducted in the absence of any commercial or financial relationships that could be construed as a potential conflict of interest.
