# Supplementary figures and images for: IL-22-Expressing Murine Lymphocytes Display Plasticity and Pathogenicity in Reporter Mice
Source: Front Immunol. 2016 Jan 19;6:662. doi: 10.3389/fimmu.2015.00662 (PMC4717188; doi:10.3389/fimmu.2015.00662)

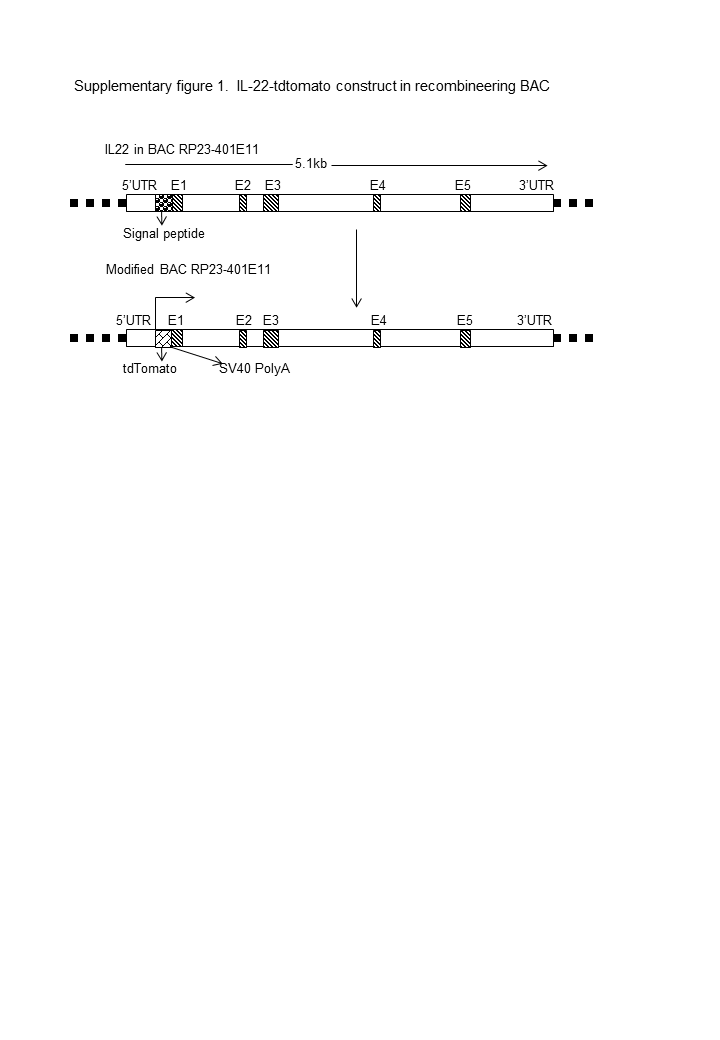

Supplement: Supplementary file 1 [file Image_1.TIF]

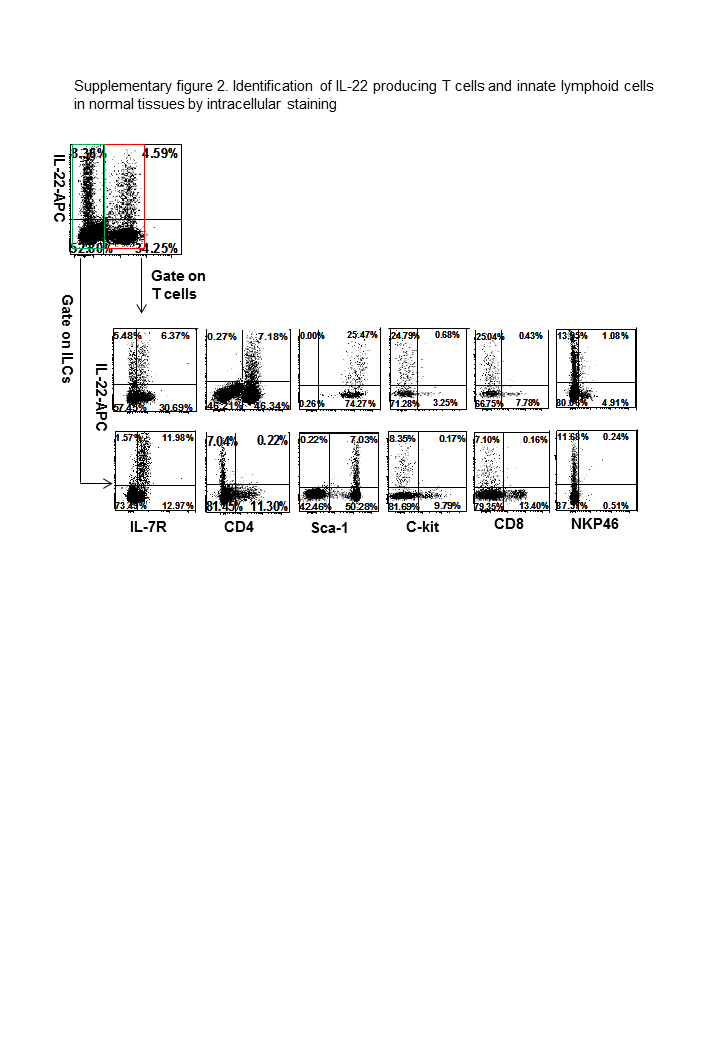

Supplement: Supplementary file 2 [file Image_2.TIF]

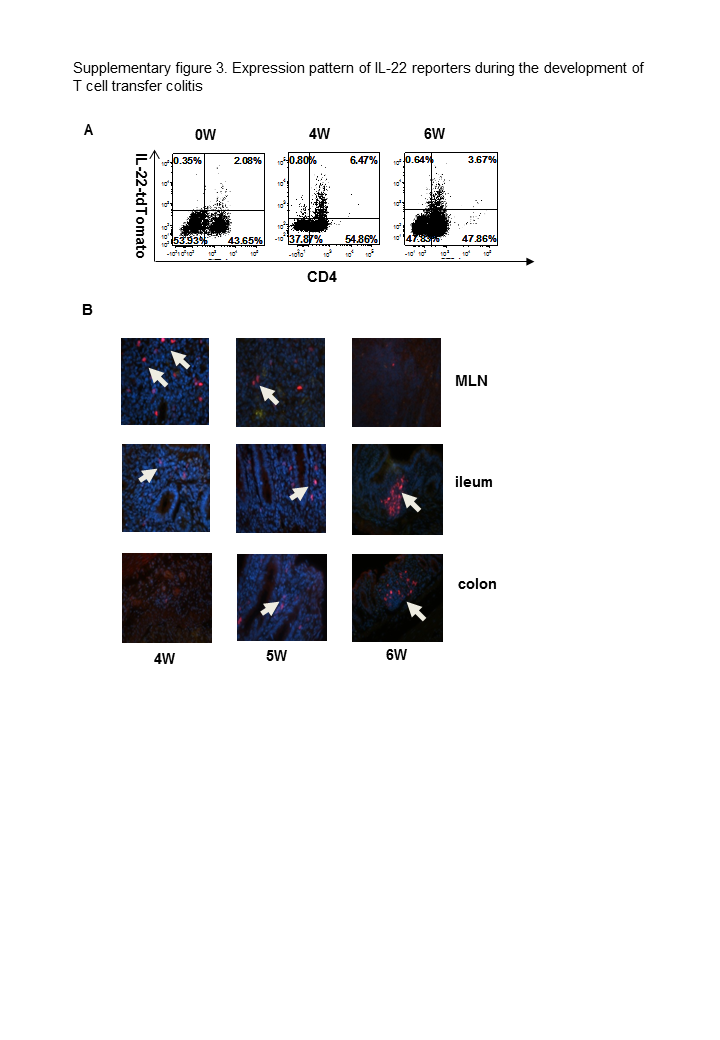

Supplement: Supplementary file 3 [file Image_3.TIF]

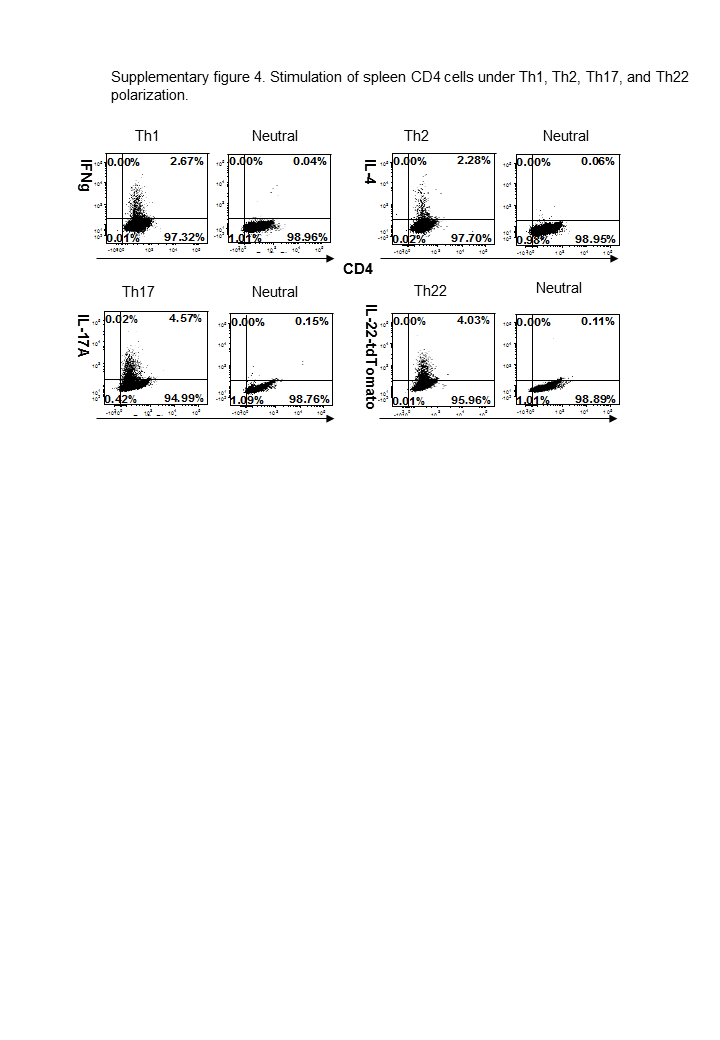

Supplement: Supplementary file 4 [file Image_4.TIF]

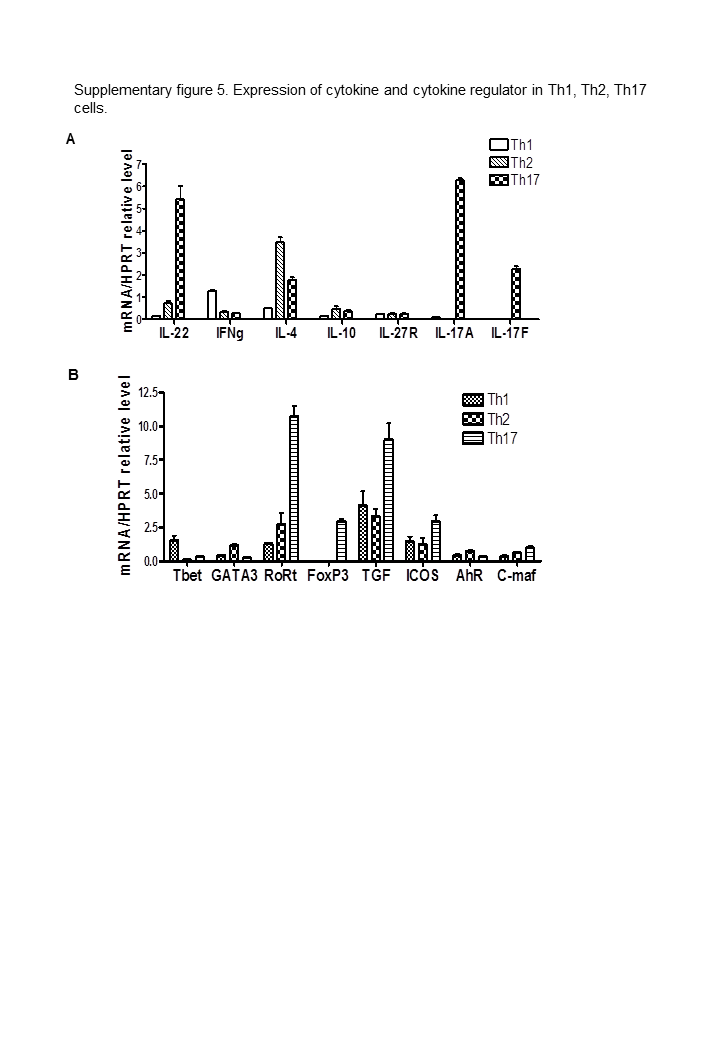

Supplement: Supplementary file 5 [file Image_5.TIF]

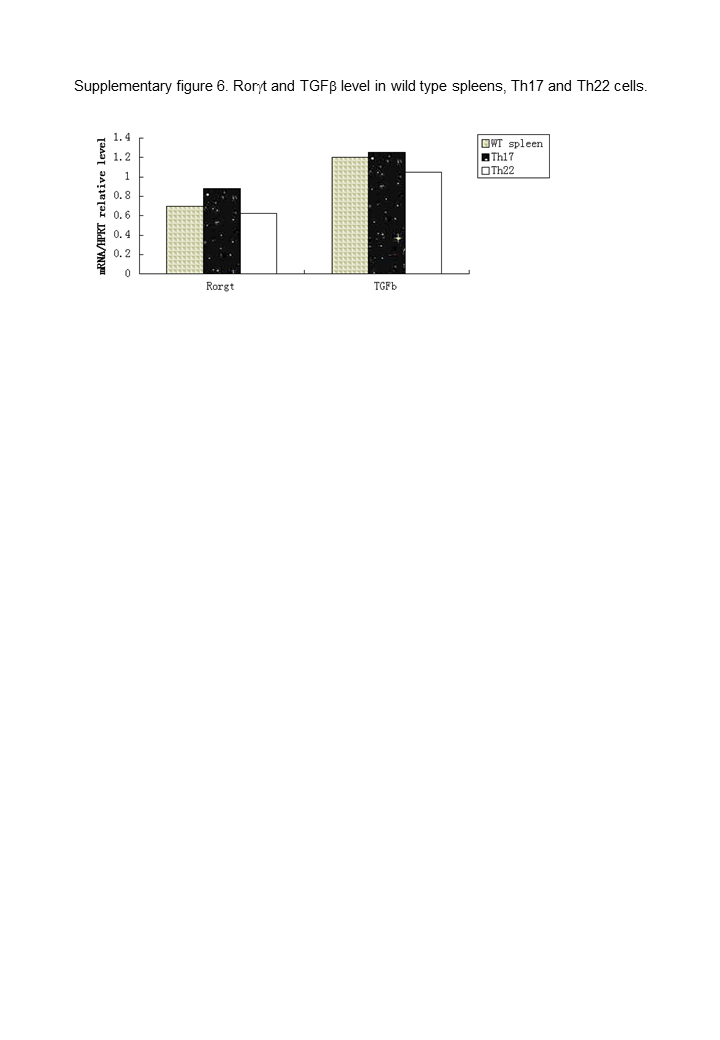

Supplement: Supplementary file 6 [file Image_6.TIF]

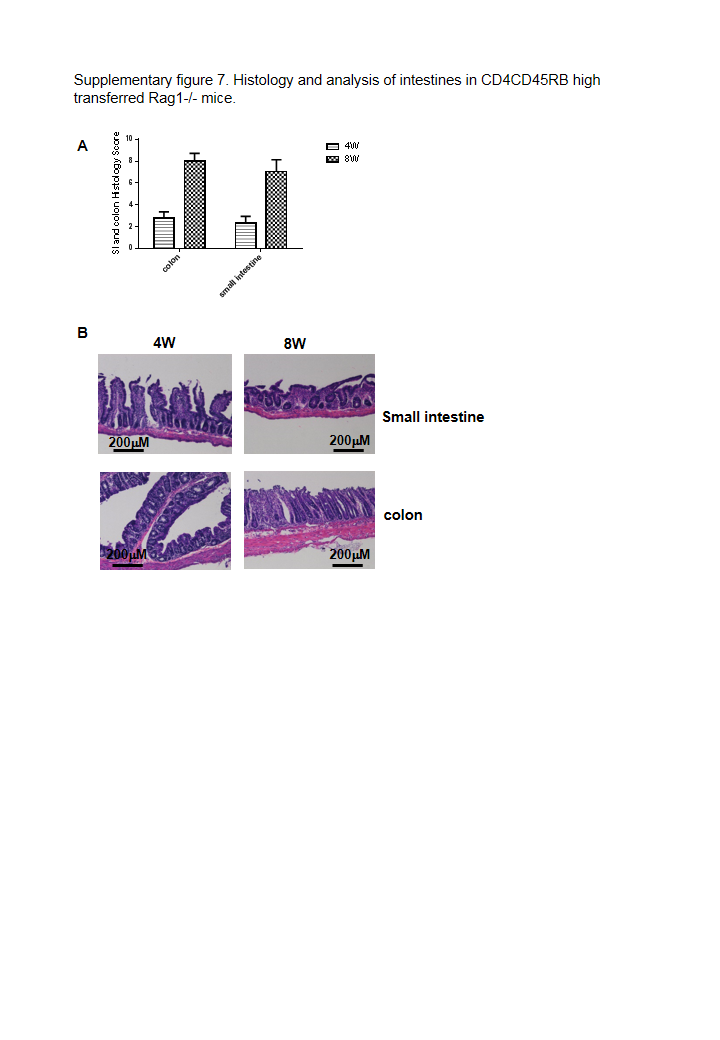

Supplement: Supplementary file 7 [file Image_7.TIF]

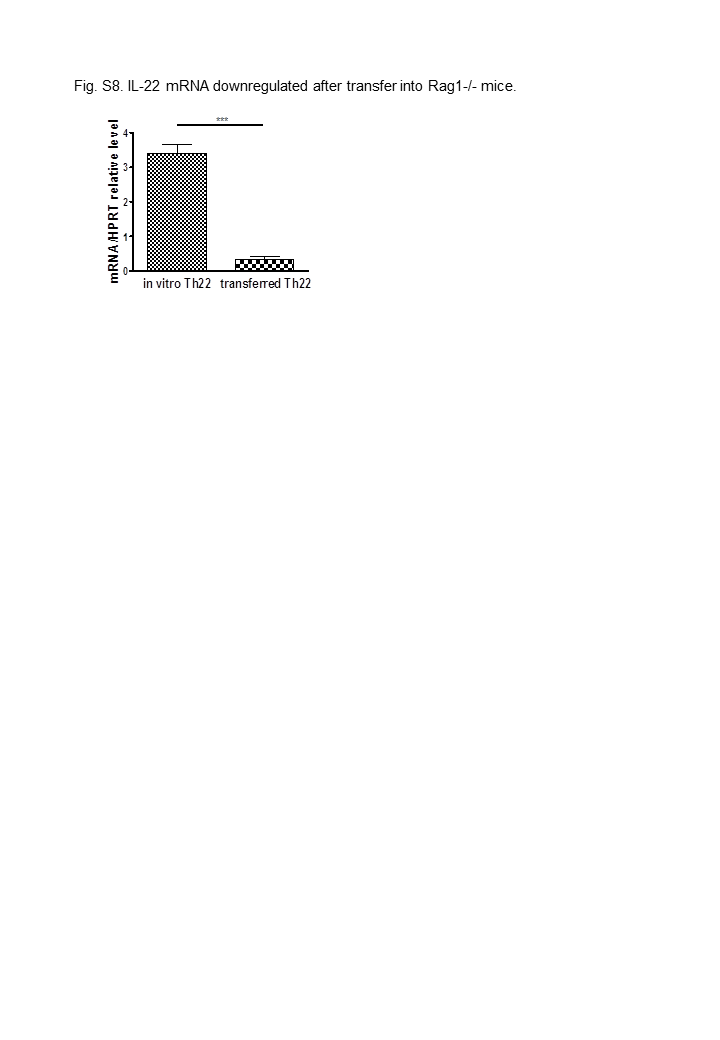

Supplement: Supplementary file 8 [file Image_8.TIF]
